# Cell Differentiation and Checkpoint

**DOI:** 10.16966/2381-3318.107

**Published:** 2015-07-02

**Authors:** Sara Cuesta Sancho, Toru Ouchi

**Affiliations:** Department of Cancer Genetics, Roswell Park Cancer Institute, Buffalo, NY14263, USA

**Keywords:** Differentiation, Checkpoints, DNA damage response, C2C12

## Abstract

DNA damage is induced in many types of cells by internal and external cell stress. When DNA is damaged, DNA Damage Response (DDR) programs are activated to repair the DNA lesions in order to preserve genomic integrity and suppress subsequent malignant transformation. Among these programs is cell cycle checkpoint that ensures cell cycle arrest and subsequent repair of the damaged DNA, apoptosis and senescence in various phases of the cell cycle. Moreover, recent studies have established the cell differentiation checkpoint, the other type of the checkpoint that is specifically activated in the course of differentiation. We will discuss the evidences that support the link between DNA damage proteins and C2C12 cell differentiation.

Genomic integrity is primordial to any organisms. It has been well illustrated that diverse stress from both intrinsic (ex. Reactive Oxygen Species (ROS)) and extrinsic (ex. ionizing radiation (IR), UV light, chemical) environment cause DNA lesions [1]. When DNA damage checkpoint is activated, proliferating cells arrest the cell cycle, allowing the damaged DNA to repair. This process is initiated by recruiting the MRN complex (MRE11-RAD50-NBS1) to DNA Double Strand Breaks (DSBs) and Single Strands Breaks (SSBs), followed by activation of Ataxia-Telangiectasia Mutated (ATM) and Ataxia-Telangiectasia and RAD3-Related (ATR), respectively. ATM and ATR phosphorylate a variety of their substrates, those including p53, MDM2, CHK2, 9-1-1- complex (RAD9, RAD1, HUS1), CHK1, etc [2–7].

Differentiation is the process in which cells become specialized from the precursor cells to specific cell type, such as neurons, lymphocytes and muscle through differentiation. A global reprogramming of gene expression and withdrawal from the cell cycle are required for the differentiation process [8]. Although it is not well understood how differentiation program proceeds under conditions of DNA damage, it is considered that it could not be completed without the repair of the DNA lesions. Therefore, it is assumed that if cells start the differentiation program prior the DNA was restored, it could lead to abnormally differentiated cells with compromised functions [9].

C2C12 cells have been widely used as an in vitro model to study myogenic differentiation process. These cells are derived from the mouse skeletal muscle C2 cell line, and they have similar characteristics to those of isolated human skeletal muscle cells [10,11]. Myogenic differentiation consists of a multistep processes that involves two major mechanisms. The first one consists of the induction of the muscle-specific genes expression by Myogenic Regulatory Factors (MRFs). MRFs induce the expression of, for example, Myf-5, MyoD, MRF4 and Myogenin. MyoD and Myf-5 which are primarily expressed in proliferating, undifferentiated myoblasts, allowing the differentiation program start, acting as a determination genes, while Myogenin expression is induced as a result of muscle differentiation (Figure 1) [12–14]. Transcriptional pathways regulated by multiple groups of muscle-specific transcription factors initiate the de novo synthesis of various muscle-specific proteins [15]. The second step in differentiation process is to make a commitment of myogenic cells to irreversible withdrawal from the cell cycle leading permanent G1 phase [16–18]. Withdrawal from the cell cycle causes morphological changes, mononucleated myoblasts alignment, and fusion of their membranes to form multinucleated myotubes, leading to the mature muscle fibers. Accomplishment of these two phases is essential for multinucleated myotubes formation.

It has been shown that during differentiation DNA Double Strand Breaks (DSBs) occur. For example, development of B lymphocytes requires the induction and consequent repair of DSBs during rearrangement of the antigen receptor genes [19]. Interestingly, there are some biochemical experiments indicating the link between modification of the DDR proteins and neuronal stem cell differentiation. IR-induced DSBs induce acetylation of p53 Lys320 in the Central Nervous System (CNS) [20,21], and acetylated p53 Lys320 promotes neurite outgrowth in vitro and axon regeneration in vivo [22]. Of note, while these results show that DSBs promote cell differentiation of B lymphocyte and neurons, DDR-regulated differentiation checkpoint has been implicated by C2C12 myoblasts, which prevents the appearance of abnormally differentiated cells [9]. Thus, it detains the progression of differentiation until DNA is repaired during muscle differentiation under conditions of genotoxic stress. After serum withdrawal when C2C12 cells were exposed to genotoxic agents, like etoposide and IR, myotube formation is blocked by cell cycle arrest followed by c-Abl-dependent inhibition of MyoD activation. This inhibition of MyoD under genotoxic stress is independent of p53 and c-Jun. c-Abl can phosphorylate MyoD at N-terminal tyrosine (Tyr30) localized within the transactivation domain. Mutations on Tyr30 and Tyr212 to Phe do not interfere with MyoD functionality but mutants become resistant to inhibition of MyoD by DNA damage. Of note, removal of these agents from C2C12 cell culture leads to repair DNA and re-induction differentiation of C2C12 cells into myotubes, indicating that differentiation checkpoint is reversible.

Not only c-Abl, but also several DDR proteins have been implicated to be involved in the differentiation checkpoint, including ATM [23–25] and NBS1 [26,27], etc. Upon DNA stress, ATM autophosphorylates its own Ser1981, leading to dimer dissociation and, subsequently, phosphorylation of H2AX and a number of transducers and effectors of DNA damage-activated pathways [28,29]. For example, ATM activates CHK2 kinase by phosphorylating its Thr68 [30–32]. Activated CHK2 coordinates a number of cellular processes by phosphorylating downstream effectors, such as CDC25A, CDC25C, BRCA1, PML1, and bRYEFp53, resulting in cell cycle arrest or apoptosis [32–34]. On the other hand, ATM directly phosphorylates p53 at Ser15, causing inhibition of p53-MDM2 interaction and promoting p53-dependent gene expression [35–37].

Larsen et al. demonstrated that this DDR pathway is activated at the early stages of differentiation of C2C12 cells [38]. Phospho H2AX (g(γ)H2AX) co-localizes to the actual site of DSBs, recruiting and/or stabilizing multiple protein complexes involved in DNA damage signaling [28–30,39,40]. They have demonstrated that, when differentiation is induced, H2AX foci appear in 12h, but most of the signals disappear in 48h. These results indicate that differentiation signals damage DNA during myoblast differentiation. C2C12 myoblasts express wild-type p53 (wtp53) protein, and it has been shown that p53 is activated during differentiation in these cells, suggesting the potential role of the protein in muscle differentiation [41–44]. This model has been supported by the results using immortal and primary myoblasts. Thus, expression of dominant negative p53 (DNp53) proteins in those cells inhibits terminal differentiation. Although it is well documented that spontaneous apoptosis occurs at myogenic differentiation, the mechanism is largely unclear [39,45–48]. Interestingly, DNp53 expression does not affect the cell cycle withdrawal and apoptotic death associated with differentiation process [41,42]. Other studies have also illustrated the possible link of p53 to muscle differentiation. Porrelo et al. have shown that p53 activated in response to DNA damage is rapidly stabilized, binding DNA to the Rb promoter, increasing its expression and inducing muscle differentiation but it is really dependent on the cell differentiation status [42]. In early differentiation (when cells are myoblasts) pRb inhibits DNA synthesis by binding to E2F resulting in repression of cyclin E/cdk2, cyclin D/cdk4 and 6, and cyclin A/cdk2 complexes [49–53]. On the other hand, when cells are in the late differentiation state (myotubes), pRb can promote differentiation binding to MyoD inducing the expression of differentiation makers like Myogenin and MRF4 [54–58] (Figure 2). These results indicate that p53 playing roles in not only inducing genes involved in growth arrest, apoptosis and DNA repair, but also regulating genes whose expression are critical for differentiation [59–62].

In summary, myogenic differentiation consists of multistep, including appearance and repair of DSBs. When DSBs are generated, DDR proteins are activated to properly ensure the DNA repair before proceeding differentiation, guarantying the correct formation of differentiated cells without compromised genome. Of note, our recent findings showed that ATM inactivation. causes insufficient generation of dendritic cells from bone [63]. Taken together, these results provide a notion that inactivation of DDR proteins results in the abrogation of differentiation.

## Figures and Tables

**Figure 1:**
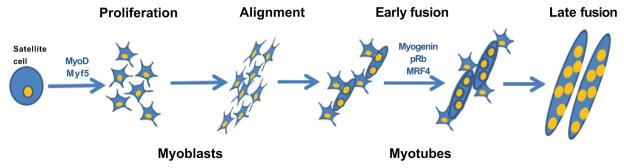
Myogenic differentiation. Satellite cells (muscle precursor cells) upon stimuli start to proliferate and differentiate into myoblasts (mononuclear cells). The myoblasts proliferate and fuse together to create myotubes over the course of several days. Additional myoblasts fuse to the existing myotubes in the late fusion step to produce larger myotubes. The differentiation process is regulated by many factors, differentiation markers changes during the course of differentiation expressing MyoD and Myf5 at the early steps of the process and Myogenin, MRF4 and pRb when the fusion already start.

**Figure 2:**
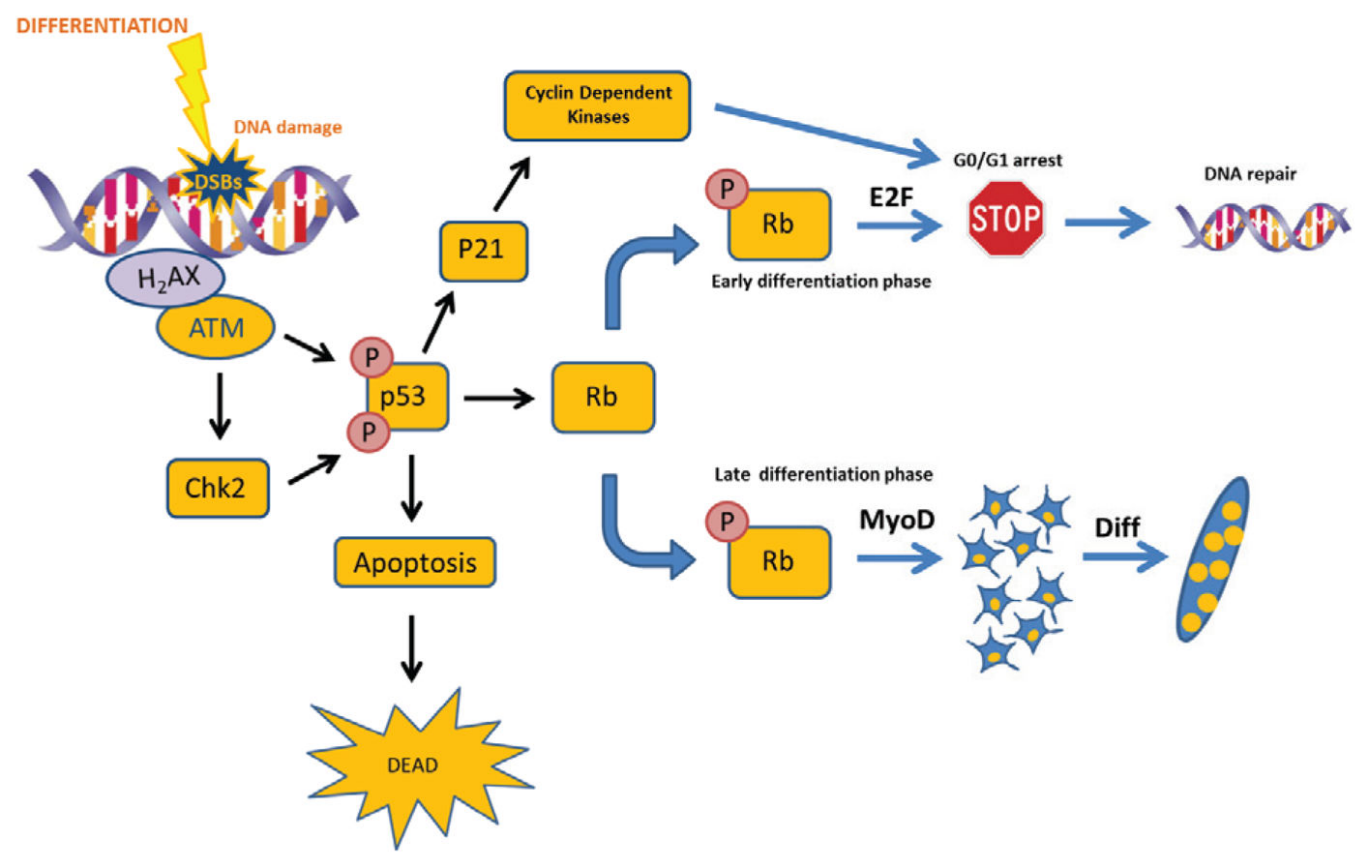
Schema explaining the role of DDR in myogenic differentiation. Differentiation generates DSBs and the DDR is activated. ATM phosphorylates p53 which could stop the cycle until the DNA damage is repaired activating p21, CDks and generating a G0/G1 arrest. If the DNA damage is not repaired, cells undergo to apoptosis. Finally, p53 plays a role in differentiation through Rb phosphorylation which could activates differentiation or stop it depending on the cell differentiation status.
